# Green synthesis and characterization of UKMRC-8 rice husk-derived mesoporous silica nanoparticle for agricultural application

**DOI:** 10.1038/s41598-022-24484-z

**Published:** 2022-11-23

**Authors:** Deivaseeno Dorairaj, Nisha Govender, Sarani Zakaria, Ratnam Wickneswari

**Affiliations:** 1 Institute for Environment and Development (LESTARI), Universiti Kebangsaan Malaysia, 43600 Bangi, Selangor Malaysia; 2 Institute of Systems Biology (INBIOSIS), Universiti Kebangsaan Malaysia, 43600 Bangi, Selangor Malaysia; 3 Department of Applied Physics, Faculty of Science and Technology, Universiti Kebangsaan Malaysia, 43600 Bangi, Selangor Malaysia; 4 Nomatech Sdn. Bhd, Faculty of Science and Technology, Universiti Kebangsaan Malaysia, 43600 Bangi, Selangor Malaysia

**Keywords:** Biological techniques, Plant sciences, Nanoscience and technology

## Abstract

Agriculture plays a crucial role in safeguarding food security, more so as the world population increases gradually. A productive agricultural system is supported by seed, soil, fertiliser and good management practices. Food productivity directly correlates to the generation of solid wastes and utilization of agrochemicals, both of which negatively impact the environment. The rice and paddy industry significantly adds to the growing menace of waste management. In low and middle-income countries, rice husk (RH) is an underutilized agro-waste discarded in landfills or burned in-situ. RH holds enormous potential in the development of value-added nanomaterials for agricultural applications. In this study, a simple and inexpensive sol–gel method is described to extract mesoporous silica nanoparticles (MSNs) from UKMRC8 RH using the bottom-up approach. RHs treated with hydrochloric acid were calcinated to obtain rice husk ash (RHA) with high silica purity (> 98% wt), as determined by the X-ray fluorescence analysis (XRF). Calcination at 650 °C for four hours in a box furnace yielded RHA that was devoid of metal impurities and organic matter. The X-ray diffraction pattern showed a broad peak at 2θ≈20–22 °C and was free from any other sharp peaks, indicating the amorphous property of the RHA. Scanning electron micrographs (SEM) showed clusters of spherically shaped uniform aggregates of silica nanoparticles (NPs) while transmission electron microscopy analysis indicated an average particle size of < 20 nm. Besides Energy Dispersive X-Ray which validated the chemical constituent of the silica NPs, the Fourier transform infrared (FT-IR) spectra showed peaks at 796.4 cm^−1^ and 1052 cm^−1^ corresponding to O–Si–O symmetric stretching vibration and O–Si–O asymmetric stretching, respectively. The Brunauer–Emmet–Teller (BET) analysis indicated an average pore size = 8.5 nm while the specific surface area and the pore volume were 300.2015 m^2^/g and 0.659078 cm^3^/g, respectively. In conclusion, agrowaste-derived MSN was synthesized using a simple and economical sol–gel method without the addition of surfactant reagents for controlled formation at the structural level. Owing to the MSNs’ excellent physical properties, the method established herein, could be used singly (without any modifications) for the functionalization of a myriad of agrochemicals.

## Introduction

Rice husk (RH) makes up 20% of the bulk grain weight of rice^1^. It is a by-product obtained at default from the paddy and rice industry. The RH dumps are often set on fire and thus, open burning emerged as the most favoured method of RH disposal^2^. Being the staple food for more than 3 billion people^3^, especially Asians, rice farming generates a substantial amount of agrowaste such as straw, husk and ash^4^. On an annual basis, it is estimated that more than 120 tonnes of RH end up as waste material after the milling process^5^. Since rice milling is essential to produce edible rice kernels of good quality^6^, the resultant waste management must as well adhere to sustainable agriculture and circular economy.

Rice husk (RH) is composed of cellulose (40–50%), lignin (25–30%), and silica (15–20%)^7,8^. Open burning of RH generates rice husk ash (RHA), a detrimental pollutant. The RHA is about 25% of RH fresh weight^1^. RH has a low nutritive content and is highly resistant to degradation. It is commonly used as a feed ingredient and low-cost burning fuel^9,10^. In others, it is a raw material valued in industrial applications for its silica-enriched property^10^. However, the downstream application of RH multi-fold with pre-processing, especially after the combustion step. The combustion step results in 90–98% of silica and trace amounts of metal oxides^11^. The silica-enriched ash is highly porous, light and displays a large external surface area^12^.

The properties and characteristics of RHA are ideal for the production of value-added materials in the following fields: ceramics, construction, semiconductor, polymer, pharmaceuticals and agriculture^13,14^. The silica RHA could either turn amorphous or crystalline, depending on the duration of combustion and temperature^15,16^. The amorphous silica RHA is composed of highly reactive silica tetrahedral arranged in a random three-dimensional network. It has a large surface area, reactive and soluble^17^. On the other hand, crystalline RHA is made of repetitive units of silica tetrahedrals arranged in a three-dimensional orientation which are inert and less reactive^18^. At an optimum temperature range of 600–700 °C, highly porous amorphous silica is obtained while at any temperature range of > 850 °C, the crystalline ash is produced^16,19^.

Green fertilizers and nanotechnology offer enormous eco-friendly solutions in sustainable agriculture. As such, the utilization of nanomaterials in precision agriculture will cut on nutrient losses and leaching during fertilization, minimize the application rate of chemical fertilizers and pesticides, increase plant nutrient use efficiency, act as soil sensors, facilitate remote sensing, harvest analysis, pest monitoring and geographical information system^20–22^. According to the Food and Drug Administration (FDA), nanoparticles (NPs) are defined as inorganic materials with less than 1000 nm dimensions. They carry useful bulk properties such as optical or magnetic properties^23^. In general, there are three types of NPs classified based on the pore size (PS) range: (i) microporous; PS up to 2 nm (ii) mesoporous; PS = 2–50 nm and (iii) macroporous; PS = 50–1000 nm. Mesoporous silica nanoparticles (MSNs) have unique and beneficial structural features, including high surface area, high pore volume, stable mesoporous structure, adjustable pore diameter, adjustable particle size, and simple internal and external surface functionalization^24–27^.

Silica NPs have long been the centre-stage of nanoparticle research especially for drug delivery and cancer research for its low toxicity and high stability ^23,28–34^, large pore volume (~ 1cm^3^/g), high surface area (> 1000 m^2^/g), ease of functionalization, low toxicity and biodegradability^35–38^. Besides the biomedical application, MSNs are used for adsorption of organic, inorganic, and gas compounds^39^, catalyst^40,41^ and enzyme immobilization^42^. Comparatively, MSNs have yet to relish its full potential in the agricultural industry. The porosity and adjustable properties such as diameter, shape, and both core and surface features of MSNs allow it to act like a cargo or a vehicle that loads small molecules or compounds, proteins and nutrients and unloads them into the soil system^23^.

The objective of the current study is to establish a green protocol for MSNs extraction from the UKMRC-8 rice husk using a simple sol–gel method under mild conditions. The synthesis procedure of MSNs with high surface area and porosity is described and characterized. Since the porosity of NPs imparts a fundamental impact on the material down- and upstream applications, the scope of MSN for agricultural application is discussed.

## Materials and methods

### Raw material

Rice husk (RH) from the UKMRC-8 rice variety was obtained from Nomatech Sdn. Bhd, Selangor Malaysia. Sodium hydroxide and hydrochloric acid were both procured from Merck (Darmstadt, Germany).

### Rice husk pre-processing and calcination

Rice husk was thoroughly washed with water to remove dirt, dust, sand and other contaminants and then dried in an oven at 100 °C for 24 h. Next, the washed RH was boiled in 1 M HCl for 2 h at 80 °C followed by multiple times of washing with distilled water to remove residual acid and other impurities. The acid-treated RH was dried at 90 °C for an overnight. Prior to calcination, the dried RH was ground to facilitate uniform combustion at different temperatures of 550 °C, 600 °C, 650 °C and 700 °C for 4 h in a programmable box furnace (Lindberg/Blue). For subsequent extraction of MSNs, only one calcination temperature corresponding to amorphous RHA (based on XRD) production was selected.

### Characterization of rice husk ash (RHA)

The phase composition and the degree of crystallinity of RHA were determined by X-ray Diffraction (XRD) (Bruker Axs D8 Advance) with a radiation source of Cu Kα of λ = 1.54060 and a scan rate of 0.025 º*θ* s^−1^ in the 2*θ* range of 5–80°. For elemental composition determination, the X-ray Fluorescence (XRF) (Rigaku, ZSX Primus IV) was used.

### Extraction of mesoporous silica nanoparticles (MSNs)

The MSN was extracted from amorphous RHA according to sol–gel method with few modifications^43,44^. Briefly, the RHA was mixed with 1 M sodium hydroxide solution at 1:10 ratio (weight/volume) and heated at 80 °C with constant stirring for 2 h until dissolved. The solution was then filtered using the Whatman no. 41 ashless filter paper and allowed to cool. Using the drop-wise technique, 1 M HCl was added to the filtrate at room temperature until pH 7 is obtained. The silica gel formed was aged at room temperature for 24 h. Distilled water was added to the gel and broken to form a slurry. The slurry was washed repeatedly with warm distilled water before drying at 110 °C for 24 h. Following drying , the formed silica was ground into fine powders and milled with a ball mill. The following equations show the reactions involved during MSNs synthesis via the sol–gel method:

$${\text{SiO}}_{{2}} \left( {{\text{ash}}} \right) + {\text{2NaOH}} \to {\text{Na}}_{{2}} {\text{SiO}}_{{3}} + {\text{H}}_{{2}} {\text{O}}$$

$${\text{Na}}_{{2}} {\text{SiO}}_{{3}} + {\text{HCl}} \to {\text{SiO}}_{{2}} + {\text{NaCl}} + {\text{H}}_{{2}} {\text{O}}$$

### Electron microscopic observation

The surface topology, morphology and elemental composition of the MSNs were characterized using a high-resolution field emission scanning electron microscope (FESEM) (Zeiss SUPRA, Germany) coupled with energy dispersive x-ray spectroscopy (EDX) (Oxford EDX INCA Penta FETX3). The structure and particle size were examined using a transmission electron microscope (TEM) (Talos L120C, Thermo Fisher Scientific, USA).

### Physical characterization

Functional groups present in the synthesized MSN were identified from the spectra of Fourier transform infrared (FTIR) (Perkin Elmer, Germany). The spectra were collected in the range of 600–4000 cm^-1^. The surface area, pore size and pore volume were assessed by BET (Brunauer–Emmett–Teller) and BJH (Barrett–Joyner–Halenda) analyses^45^. The nitrogen gas adsorption–desorption analysis was performed using ASAP 2020 from Micromeritics at 77 K. Samples were degassed under vacuum at 573 K for three hours before the analysis^9^.

## Results

### Characterization of rice husk ash (RHA)

**Figure 1:**
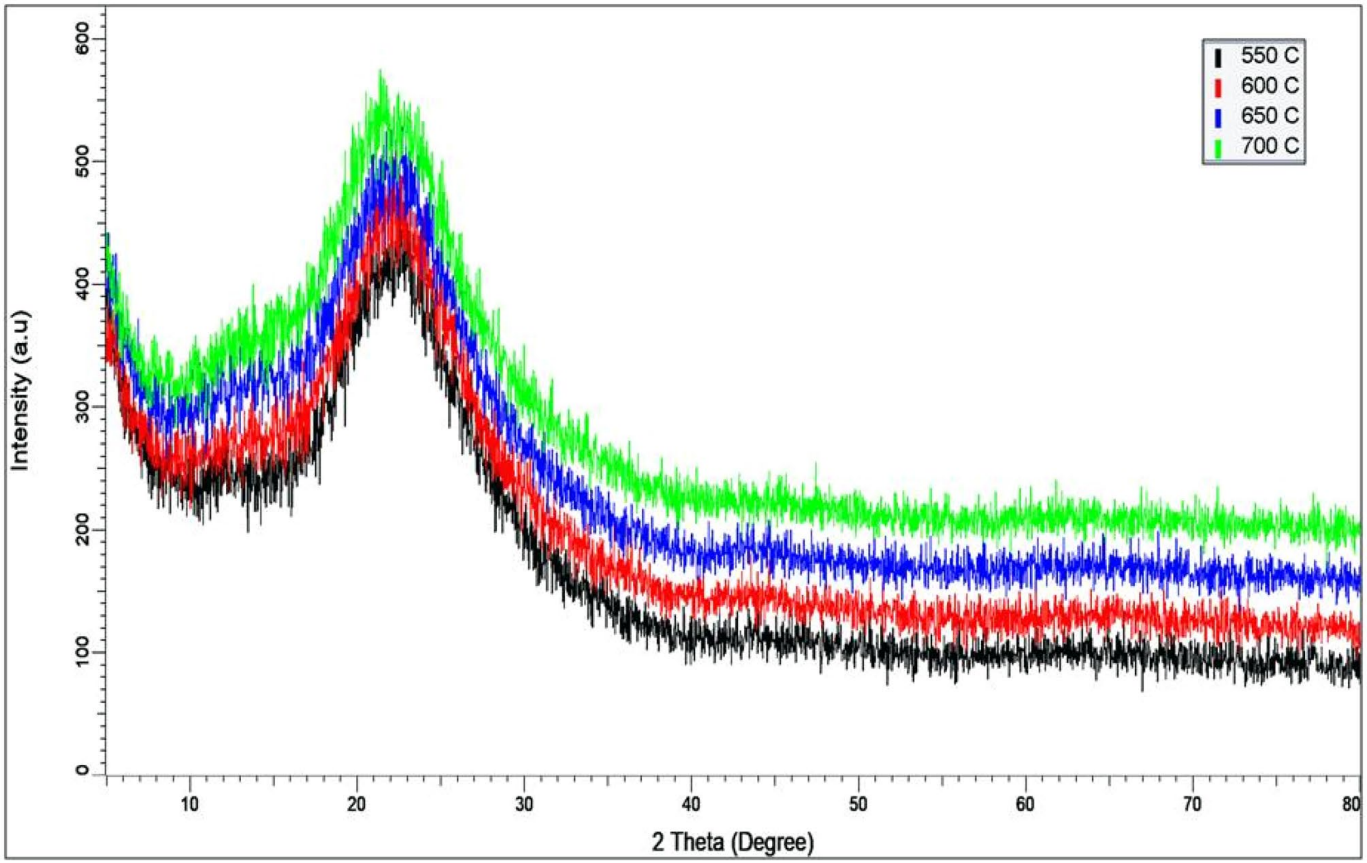
X-ray diffraction (XRD) spectra of rice husk ash calcined at four different temperatures.

Results as shown by the broad peak around 2θ≈20–22 °C indicated that all four different calcination temperatures selected in this study feasibly produced amorphous RHA (Fig. 1). This was further confirmed by the absence of any sharp peaks which correspond to a well-ordered crystalline state. Hence, for the synthesis and characterization of NPs, a calcination temperature of 650 °C was selected throughout the method. The X-ray fluorescence analysis revealed a slight increase in silica content (more than 2%) after calcination (Table 1). Likewise, the Si composition in RHA showed a 6% increase from 91.2% in acid-leached RH.

**Table 1:** X-ray fluorescent analysis showing the chemical composition of acid leached UKMRC-8 rice husk and rice husk ash.

Component	Rice husk (wt%)	Rice husk ash (wt%)
SiO_2_	96.2	98.4
Cl	1.22	–
SO_3_	0.01	< 0.01
P_2_O_5_	0.01	< 0.01
Fe_2_O_3_	< 0.01	0.01
Cr_2_O_3_	< 0.01	< 0.01
Al_2_O_3_	< 0.01	< 0.01
K_2_O	< 0.01	< 0.01
CaO	< 0.01	< 0.01
NiO	< 0.01	< 0.01
MgO	< 0.01	< 0.01
MnO	< 0.01	–

### The rice husk ash (RHA) to mesoporous silica nanoparticles (MSN) conversion: physical appearances and stages

**Figure 2:**
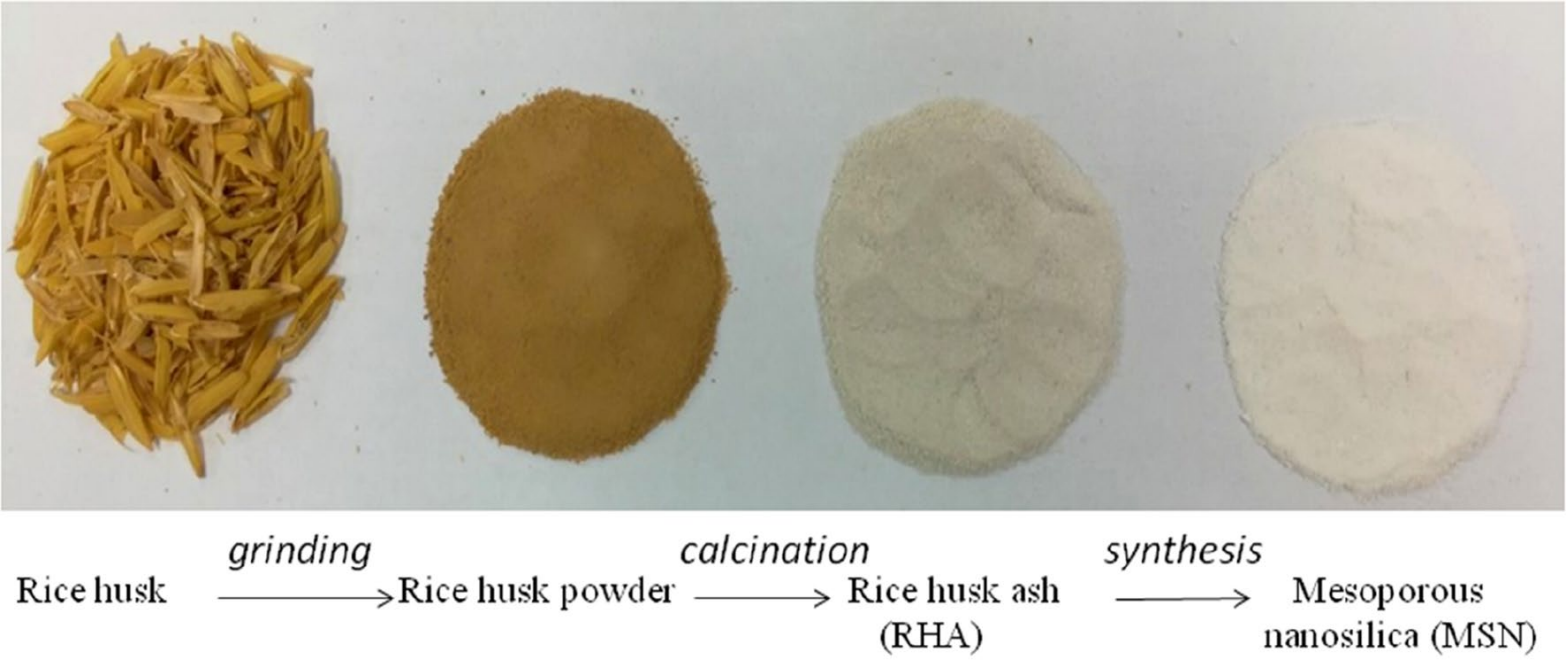
Physical appearance of the different stages of mesoporous nanosilica synthesis.

The various forms of physical transitions, from rice husk into white MSN during a simple and economical sol–gel synthesis procedure (Fig. 2).

### Electron microscopic observation

**Figure 3:**
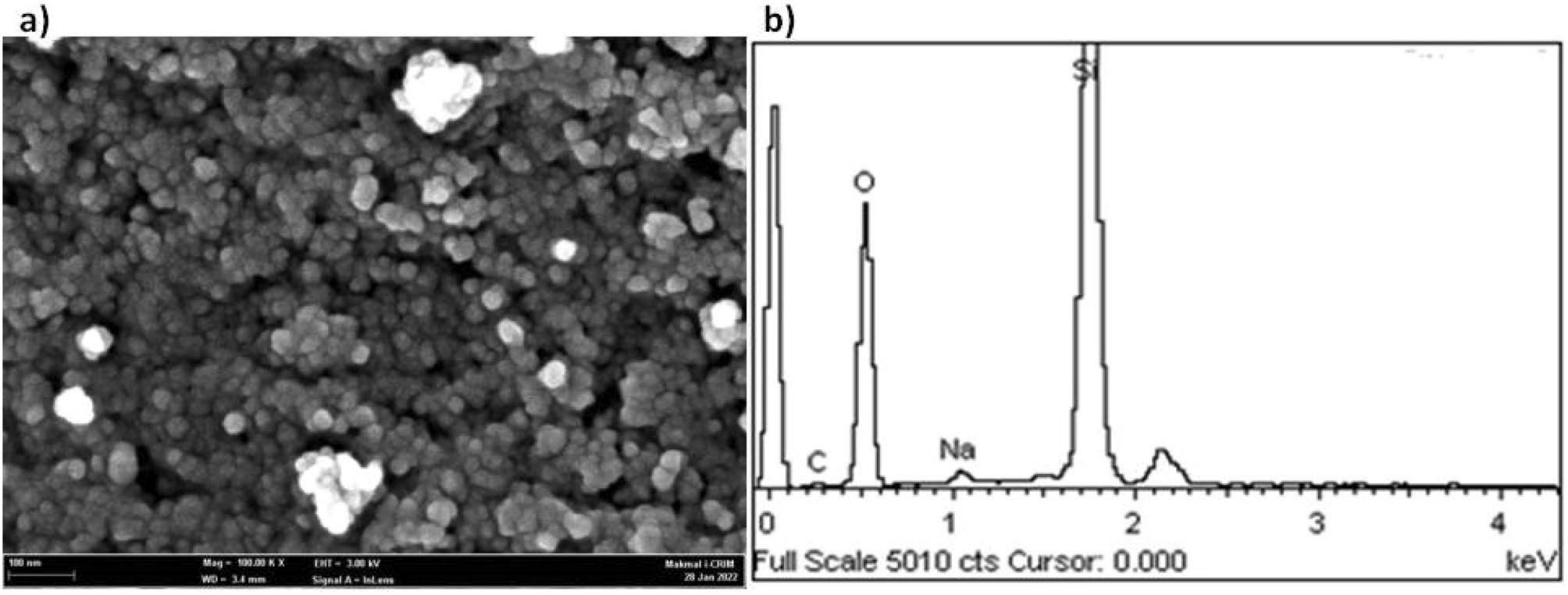
(**a**) FESEM micrograph and (**b**) EDX spectra of silica nanoparticles synthesized from UKMRC-8 rice husk ash.

**Figure 4:**
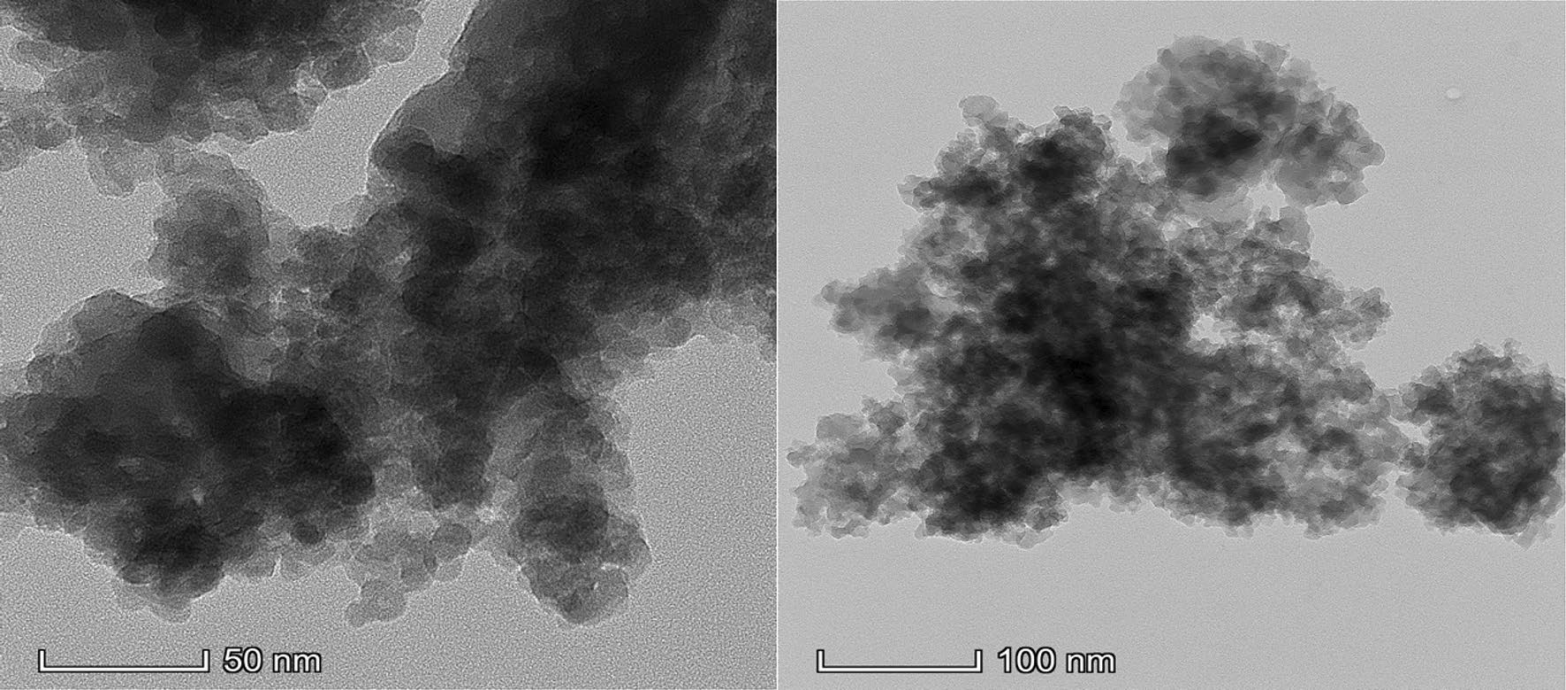
Transmission electron micrographs of silica nanoparticles synthesized from UKMRC-8 rice husk ash, viewed at various resolutions.

Based on FESEM visualization, the silica nanoparticles (NPs) were highly uniform and spherical with a size ranging between 15 and 25 nm (Fig. 3). The clusters of particles denote aggregation of MSNs. As for EDX analysis, besides silica contributes more than 95% of the total weight, impurities in the form of carbon and sodium were present at 3% and 1%, respectively. Further visualization with TEM shows that the size of silica NPs is < 20 nm, in alignment with the mesoporous dimension (Fig. 4).

### Spectroscopic analysis and pore size characterization

**Figure 5:**
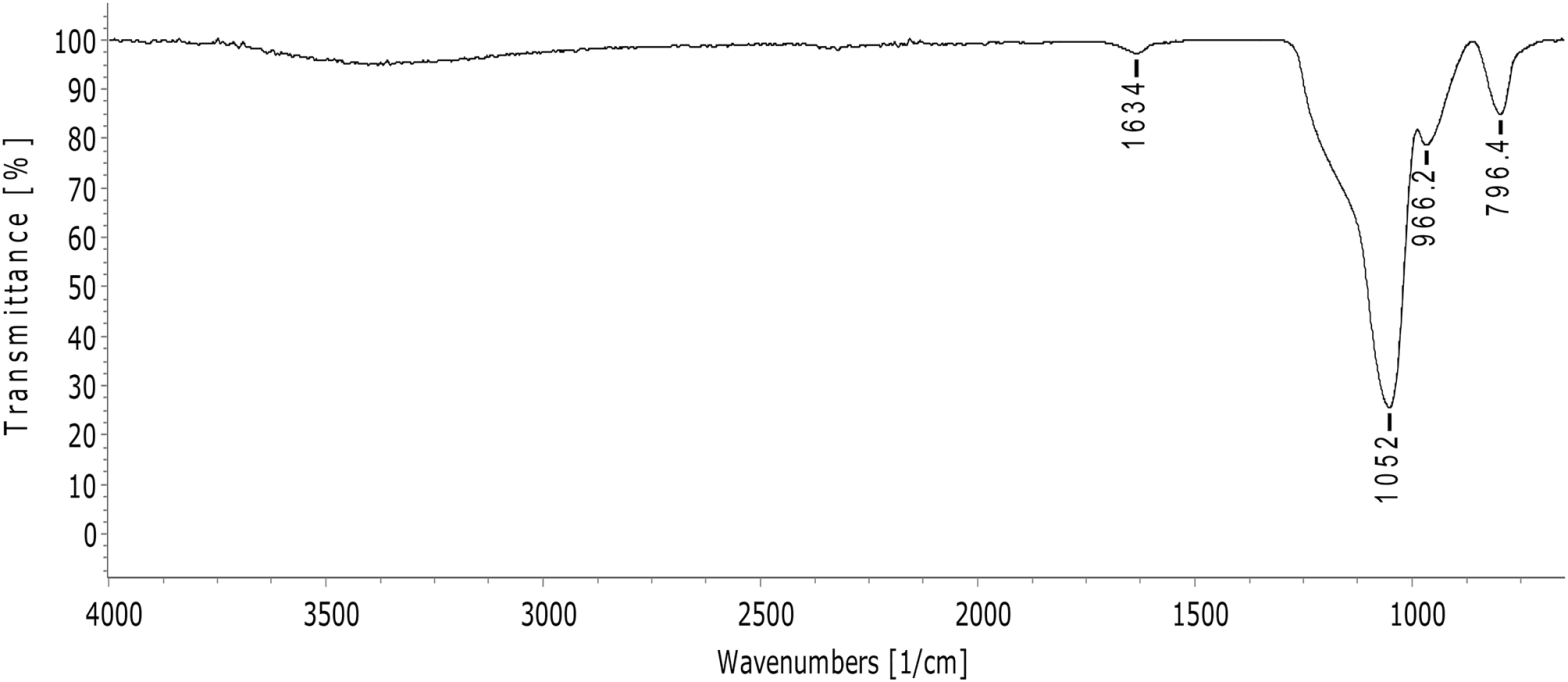
FT-IR spectrum of silica nanoparticles synthesized from UKMRC-8 rice husk ash.

The FT-IR spectrum exhibited characteristic peaks of silica. The synthesized silica NPs show peaks at 796.4 cm^−1^ and 1052 cm^−1^ corresponding to O–Si–O symmetric stretching vibration and O–Si–O asymmetric stretching, respectively (Fig. 5). Meanwhile, the strong peak at 966.2 cm^−1^ informs bending and stretching vibrations of Si–OH bonds. The peak at 1634 cm^−1^ denote the characteristic H–O–H bond vibration of water molecule.

**Figure 6:**
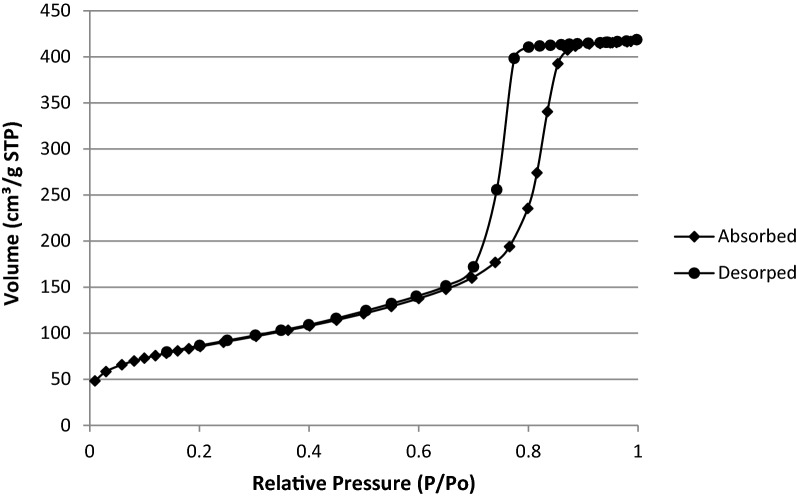
Nitrogen adsorption–desorption isotherms of silica nanoparticles synthesized from UKMRC-8 rice husk ash.

**Figure 7:**
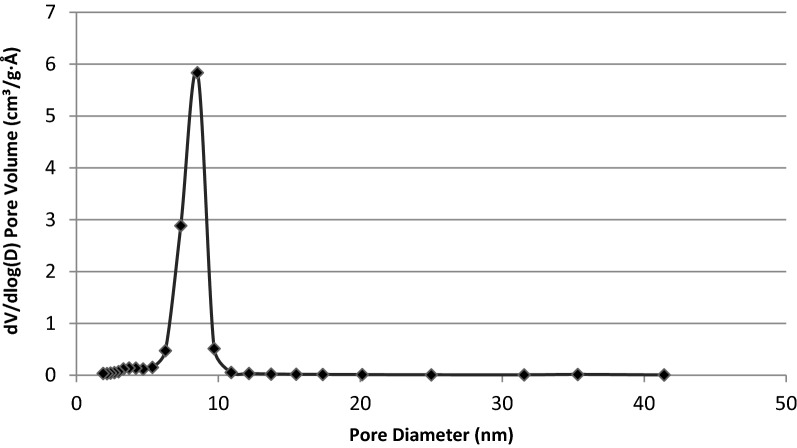
Pore size distribution of silica nanoparticles synthesized from UKMRC-8 rice husk ash.

**Figure 8:**
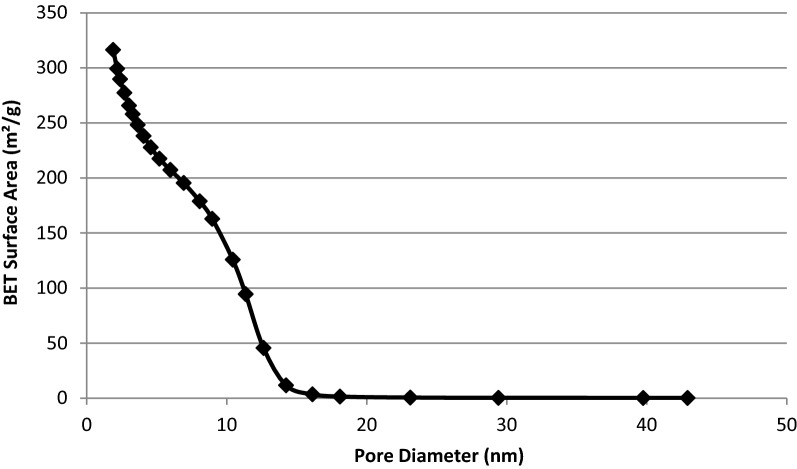
Brunauer–Emmet–Teller (BET) surface area distribution of silica nanoparticles synthesized from UKMRC-8 rice husk ash.

The nitrogen adsorption–desorption isotherm analysis displayed type IV isotherm, indicating that the synthesized silica nanoparticles were indeed mesoporous and these mesopores were filled through capillary condensation (Fig. 6). Besides, the MSN followed H1 hysteresis which is associated with porous materials, which consist of almost uniform agglomerated spheres. The average pore size was 8.5 nm, hence the synthesized silica nanoparticles were mesoporous while the BET specific surface area and the pore volume were 300.2015 m^2^/g and 0.659078 cm^3^/g, respectively (Figs. 7, 8).

## Discussion

Agricultural biomass makes up about 15% of the total agro-waste generated in Asia^46^. The paddy and rice industry generates about 0.5 million metric tonnes of rice husk annually^47^ while the daily production of agro-waste is projected to reach 0.210 (kg/capita/day) by 2025^48^. Urbanization, population growth, industrialization and changing consumption patterns demand landfill site expansion to accommodate the increasingly growing agro-waste^49^. As suitable lands become scarce, waste management is integral to minimize the negative impacts on the environment while taking mitigation measures in global warming. In addition, the perpetually growing waste disposal will put a dent on the nation’s coffers which otherwise could be channelled to better use. In most developing countries, the non-existent guidelines on the potential uses of rice husk (RH) leads to an avalanche of environmental issues resulting from open burning^50^. Although, there are several agro-waste-based products such as nanoparticles, organic fertilizer, biochar and biogas^51^, the opportunity for RH exploitation remains limited in the agriculture industry.

In this study, UKMRC-8 RH was utilized as the raw material for mesoporous silica nanoparticle (MSN) extraction. UKMRC-8 is a high-yielding rice variety (average yield = 7–8 tonne metrics per hectare) with fairly good resistance to lodging, flooding, pest and pathogen. In Malaysia, UKMRC-8 was registered as a rice planting material with variety status (PBR 0031) in 2014. It is a cross between the wild rice, (*Oryza rufipogon*) and commercial variety, MR219 (*O. sativa*) (https://www.nomatech.com.my). Rice is a high silicon (Si) accumulator and the RH constitutes about 15–20% of Si. The combustion of UKMRC-8 RH resulted to about 23–25% of ash from its fresh weight. Silica made the bulk of RHA at more than 98%. High purity RHA (98.4%) was obtained from acid treated RH. Apparently, the UKMRC-8 RH inherit high silica composition, which may be attributing to its agronomy features related to Si-mediated resistance: lodging and resistance against biotic factors. The UKMRC-8 RH is an excellent silica feedstock. The acid-leached RH were free from volatile matters, metal oxides and impurities, and organic compounds such as cellulose and lignin^7,52^. Pre-treatment of RH with acid has been reported to increase the silica purity of RHA. The results were comparable to a previous study which had reported about 95–99% of Si in RHA of local origin^1^.

Besides purity, amorphous RHA is much preferred as compared to the crystalline state as the first is highly reactive, non-hazardous and can be readily taken up by plants^53^. The structural change (extent of amorphous/crystalline) in RH is affected by the duration of incineration and incineration temperature. Thus, the combustion criteria is crucial to produce reactive silica^54,55^. The XRD analysis confirmed that the calcined RHs (RHAs) were amorphous at all four different temperatures (550 °C, 600 °C, 650 °C and 700 °C) but differed in colouration. The RH burned at 550 °C formed a greyish RHA while the RHA produced via combustion at 600 °C was ivory in colour.

For the extraction of MSNs, the fairly white RHA produced from calcination at 650 °C was used as the precursor material. In a previous study, RHA colouration has been associated with Si purity; lighter colour RHA correlates to higher purity whereas a darker RHA confers lower purity^56^. Previous studies reported that amorphous RHAs are produced at 550–800 °C while at higher temperatures (> 800 °C), the RHA takes up the crystalline form^18,57,58^. Others found that amorphous RHA can be produced at up to a combustion temperature of 700 °C while further increase at > 850 °C, assumes crystalline form^5,16,19,59^.

Silica nanoparticles (Si-NPs) are synthesized using various methods such as chemical vapour deposition, plasma synthesis, combustion synthesis, vapour-phase reaction, sol–gel processing, microemulsion, hydrothermal technique and thermo-decomposition^60–67^. The different synthesis strategies uniquely affect the particle size, surface area, pore size and shape of the Si–NPs^68,69^. In the current study, alkaline extraction (NaOH) followed by acid precipitation was carried out to recover Si-NPs from RHA under a mild condition^43^. Silica dissolves in an alkaline medium (pH > 10) to form a sodium silicate solution (pH > 12). As both the processes of hydrolysis and condensation occur simultaneously, the sol of sodium silicate converts into a polymeric network of gel in the presence of HCl that acted as the catalyst from which silica was precipitated^70^. The FTIR spectrum indicated the presence of a silica functional group and was free from other peaks which could potently correspond to other organic and inorganic functional groups of residual reagents.

Electron microscopic observation confirmed the formation of Si-NPs as aggregating clusters of uniform nanostructures. Further examination through FESEM micrographs showed that the NPs were 20–25 nm, in size. On the other hand, the TEM analysis further confirmed that the Si-NPs were mesoporous (size < 20 nm). The results were in agreement with previous studies which had demonstrated the size of mesoporous Si–NPs at < 50 nm dimension: NP size; 10–30 nm^52^, and NP size; 20–50 nm^71,72^. Besides, EDX analysis ascertained that the Si-NPs were highly pure at Si > 95% wt. The BET analysis further confirmed that the Si was mesoporous as the average pore size (PS) was 8.5 nm while BET surface area was recorded in the region of 300 m^2^/g. Comparatively, the MSNs obtained had a much smaller PS and higher surface area than the previous reports by Bhupinder^8^ (270 m^2^/g), Sankar et al.^71^ (201–247 m^2^/g) and Dhaneswara et al.^9^ (203 m^2^/g).

The primary challenge in the synthesis of Si-NPs from RHA is to obtain the desired physical properties. Highly porous and lightweight MSNs with a high external surface area^73^ are most feasible in various agro-application. These physical properties are highly advantageous in post-functionalisation applications^74^; encapsulation of agrochemicals (standard fertilizer and pesticides), soil conditioner to improve the soil quality and water retention capacity, Si fertilizer with a positive effect on plant growth and development, defense response against biotic and abiotic factors and enhanced productivity^22,75–77^. Silicon is a beneficial element for plants as it promotes mechanical and cell wall strengthening of plant structures and is not detrimental if taken in excess^78^. In rice and other cereal crops, silicon fertilization is known to increase photosynthesis, decrease susceptibility to disease and insect damage, prevent lodging, and alleviate water and various mineral stresses^76–81^. In addition, Si–NPs could also improve soil microbiome diversity^82^.

In numerous agricultural crops, Si nutrition is integrated for soil fertility and, plant growth and development enhancement. Despite being a micronutrient, Si is catered as an additional input besides the primary (N, P, K) and secondary nutrient (Ca, Mg and S) applications. In modern agro-systems, various fertilizers and fertilizing products are applied judiciously: organic and organo-mineral fertilizers, mineral fertilizers, inhibitors (improves nutrient use efficiency), liming materials (acidic soil corrective agent), organic soil improvers (soil water retention, soil physical structure and increase organic matter) and plant bio-stimulants (converts nutrient into plant available form, accelerate growth, improve tolerance to stresses and crop quality)^83,84^. A balanced plant nutrition is key towards production, nevertheless, the heavy reliance on agrochemicals to increase yield and subsequent productivity poses adverse effects to the environment and the engaging agro-system. As such, synthetic fertilizers especially nitrogenous compound supplied into soil system, leaches rapidly to cause environmental problems such as flocculation under limited rain condition (sodium nitrate), increase in soil acidity with continuous use (ammonium sulfate, ammonium nitrate), and contamination of water bodies resulting from high hygroscopic nature (urea, ammonium nitrate).

In general, Si-NPs could ideally address the high solubility and poor soil retention problem observed in the modern-day agrochemicals. Under this context, MSN could be employed as a delivery vehicle for the loading and unloading of a target agrochemical. MSN are stable, rigid and has a tuneable surface chemistry. MSNs formed through self-assembly process are often obtained via the addition of surfactants such as cetryltrimethylammonium bromide, trimethybenzene, 3-chloropropyltrimethoxysilane, tetraethylenepentamine (TEPA), tria(2-aminoethyl)amine (TREN), sodium floride and acetic acid. The different types of surfactants along the structure-directing agents positively affect the type of MSN structure formed^74,77,85^. The structure control through surfactant modification is perceived deemed chemically demanding. PS is the utmost critical feature manipulated for loading and release of a target compound. Traditionally, PS are adjusted by surfactant templates using either a long chain polymer or via the addition of a swelling agent. In numerous previous studies, the PS of MSN was expanded using amphiphilic block copolymers and 1,3,5-trimethylbenzene (most common pore expanding agent)^86^. The loading and release of agro-related compounds such as antibiotics, secondary metabolites, macronutrient (urea: MW; 60.06 g/mol, calcium carbonate; 100.1 g/mol, ammonium nitrate; 80.043 g/mol, ammonium sulphate; 132.14 g/mol, muriate of potash; 74.55 g/mol) and micronutrient are feasible for immobilization with MSN of PS < 10 nm, as demonstrated in the present findings. For example, MSN with a PS of 5.7 nm has demonstrated good ibuprofen (MW: 206.29 g/mol) loading capacity whereas a MSN with PS = 6 nm, effectively loads RNAse^87^.

Circular economy not only promotes sustainable waste management, but may also re-valorise by-products from agro-systems. In this study, the silica enriched UKMRC-8 RH, applied as a raw material for an economical synthesis of MSNs shows good potentials in a wide field of agro-applications. The MSN extraction from RHA omits surfactant modification and delivers a relatively simple procedure, inclusive of a pre-treatment step. The method is cost-effective as the reagents required are mainly inexpensive chemicals. The proposed method poses minimal environmental impact and showed high reproducibility.

## Data Availability

All data analysed in this study are included in this published article.
